# High frequency of toxigenic *Clostridium difficile* and *Clostridium perfringens* coinfection among diarrheic patients at health care facility-onset (HCFO) and community-onset (CO) centers in Bogotá, Colombia

**DOI:** 10.1186/s13099-019-0308-7

**Published:** 2019-06-03

**Authors:** Alex J. Forero, Marina Muñoz, Milena Camargo, Sara C. Soto-De León, Dora I. Ríos-Chaparro, Claudia Birchenall, Darío Pinilla, Juan M. Pardo, Diego F. Josa, Manuel A. Patarroyo, Juan D. Ramírez

**Affiliations:** 1 Grupo de Investigaciones Microbiológicas–UR (GIMUR), Programa de Biología, Facultad de Ciencias Naturales y Matemáticas, Universidad del Rosario, Carrera 24 # 63C - 69, Bogotá, Colombia; 2 Especialización en Microbiología Médica, Pontificia Universidad Javeriana, Bogotá, Colombia; 3 Posgrado Interfacultades Doctorado en Biotecnología, Facultad de Ciencias, Universidad Nacional de Colombia, Bogotá, Colombia; 4 Molecular Biology and Immunology Department, Fundación Instituto de Inmunología de Colombia (FIDIC), Bogotá, Colombia; 5 Laboratorio de Investigación Biomédica y Biología Molecular, Facultad de Ciencias de la Salud, Universidad del Sinú, Montería, Córdoba Colombia; 6 Hospital Universitario Mayor-Méderi, Bogotá, Colombia; 7 Fundación Clínica Shaio, Bogotá, Colombia; 8 School of Medicine and Health Sciences, Universidad del Rosario, Bogotá, Colombia

**Keywords:** *Clostridium perfringens*, *Clostridium difficile*, Diarrhea, Community-onset, Health care facility onset

## Abstract

**Background:**

The aim of this study was to evaluate the frequency of toxigenic *C. difficile* and *C. perfringens* infections at health care facility-onset (HCFO) and community-onset (CO), in two health care centers (HCC) in Bogotá, Colombia. A total of 220 stool samples from patients presenting diarrhea acquired at HCFO or CO were analyzed by several PCR tests.

**Results:**

We found that 65.5% (n = 144) of the population had *C. difficile* infection, followed by toxigenic *C. difficile* with 57.3% (n = 126), and finally toxigenic *C. perfringens* with a frequency of 32.7% (n = 72).

**Conclusions:**

This study is the first molecular detection and characterization of *C. difficile* and *C. perfringens* in HCFO and CO in Latin America and demonstrates a relevant frequency of these two species, including coinfection and strikingly diverse toxigenic profiles, especially in the CO.

## Introduction

*Clostridium difficile* is one of the most studied clostridial species, as it leads to developing diarrhea associated with the use of antibiotics at the hospital level [1]. The main virulence factors of *C. difficile* are Toxin A (TcdA) and Toxin B (TcdB), belonging to the large family of Clostridial toxins with glucosyltransferase activity [2]. These toxins are encoded by genes located in a region of the chromosome of approximately 20 Kb, which constitutes the pathogenicity locus (*PaLoc*). Some strains of *C. difficile* can produce a third toxin called binary, which is encoded by a chromosomal region called *CdtLoc*, located downstream of *PaLoc*, which contains the *cdtA* and *cdtB* genes, coding for its two components, in addition to a regulator for these genes (*cdtR*) [3].

On the other hand, diarrhea can also be caused by *C. perfringens*, a species that is widely distributed in various hosts and environments, and that has been related to histotoxic and intestinal infections in animals and humans. *C. perfringens* has been identified in humans as the main etiologic agent of gas gangrene, also being able to cause other complications such as diarrheal disease associated with food poisoning, necrotizing enteritis, and other nonspecific gastrointestinal manifestations [4]. Historically, it has been considered that *C. perfringens* produces the following four main toxins: alpha (CPA), beta (CPB), epsilon (ETX), and iota (ITX). However, recently some authors have added two more toxins to its toxin repertoire: enterotoxin (CPE), and necrotic enteritis B-like (NetB) toxin; all these toxins can be differentially produced [5] and determine the clinical spectrum of infection by this species [6]. CPA is recognized as the main virulence factor in humans, causing hemolytic and dermonecrotic effects, characteristic of clostridial myonecrosis, which can be lethal [5]. Different gene regions coding for the above toxins have been previously implemented for *C. perfringens* molecular detection. The *cpa* gene encoding for CPA, has been described as the best molecular target, which is located in a stable region of the genome present in the seven identified toxinotypes (A–G), according to the most recent reclassification of the species [6].

The importance of these two pathogens in terms of public health mediated by the broad toxigenic arsenal, leads to the need to evaluate the coinfection frequency (defined as a positive result simultaneously for the two species). In this context, this study aimed at determining the frequency of *C. perfringens* and *C. difficile* (any type or toxigenic specifically) present in health care facility-onset (HCFO) or community-onset (CO) diarrhea, at two health care centers in Bogotá, Colombia.

## Methods

### Study population

A total of 220 stool samples from patients with diarrhea [7], were collected during the period from September 2015 to April 2017 at two health care centers (HCC), located in the city of Bogotá, Colombia [the Hospital Universitario Mayor—Méderi (HCC-1) and the Fundación Clínica Shaio (HCC-2)]. Participant selection (HCFO and CO groups) was carried out following the guidelines of the Society for Healthcare Epidemiology of America and the Infectious Diseases Society of America [7].

### Molecular detection and toxinotyping of *C. difficile* and *C. perfringens*

The clostridial species were identified using several conventional polymerase chain reaction (PCR) tests. Two sets of consensus primers targeting constitutive genes coding for the 16S ribosomal subunit (rRNA-16S) and for the glutamate dehydrogenase enzyme (GDH) as reported elsewhere [8, 9] were initially used for *C. difficile* detection (toxigenic or non-toxigenic). Subsequently, the toxigenic profiles of the *C. difficile* positive samples were determined using six independent amplification tests, four of them directed to the *PaLoc* regions that code for the main toxins of *C. difficile* [10, 11], and the other two to the *CdtLoc*, where the coding regions for the binary toxin are located [12]. A positive result for any of these genes led to the assignment in the ‘tox_*C. difficile’* category. In the case of *C. perfringens*, the detection was carried out by conventional PCR directed to the *cpa* gene, as reported elsewhere [13], considered as an indicator of the presence of ‘tox_*C. perfringens*’.

### Statistical analyses

Descriptive analyses were carried out to determine the frequencies in terms of percentages with respect to the total population, for each event of interest. χ^2^ tests were performed to identify potential associations between the variables analyzed. A binomial logistic regression analysis was used to estimate the association between infection by *C. perfringens*, *C. difficile* or tox_*C. difficile* (taken as dependent variables) and the different factors evaluated (hospital center and hospital stay) taken as independent variables within the analysis. Additionally, the strength of association between the existing coinfections (*C. perfringens* and *C. difficile*, *C. perfringens* and tox_*C. difficile*) was calculated using odds ratios (ORs) with their corresponding 95% confidence intervals (CIs). The adjustment of the ORs (AdOR) was carried out from hospital (HCC-1 and HCC-2) and place of stay (HCFO and CO), as confounding variables. All analyses were performed using STATA14^®^ (StataCorp LLC, College Station, TX, USA). The level of significance was established at p < 0.05.

## Results

### Frequency of *C. difficile/C. perfringens* infection and/or coinfection

**Fig. 1:**
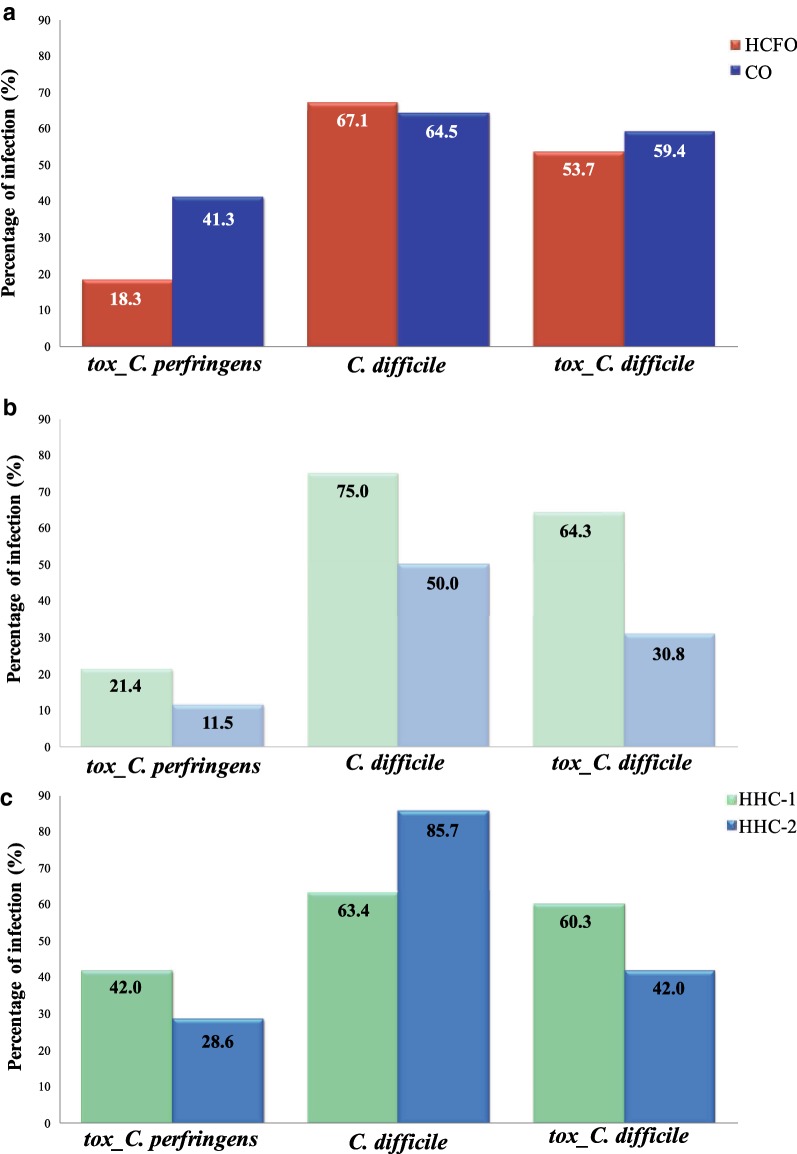
Frequency of infection of the Clostridial species evaluated. **a** In the Global population; **b** in HCFO and **c** in CO

A total of 85.0% (n = 187) of stool samples from patients with diarrhea collected for this study came from HCC-1, and the remaining 15.0% (n = 33) were from HCC-2. Of the total samples collected in HCC-1, the majority were obtained from CO patients (70.0%, n = 131), while in the case of HCC-2, the majority came from HCFO patients (78.8%, n = 26). Regarding the distribution of the evaluated species, the results showed that 65.5% (n = 144) of the population had *C. difficile* infection, followed by tox_*C. difficile* with 57.3% (n = 126), and finally tox_*C. perfringens* with a frequency of 32.7% (n = 72). When evaluating the distribution of the frequency of the species according to the stay (HCFO and CO), the frequency of *C. difficile* was higher in patients coming from HCFO compared to those of CO (67.1% and 64.5%, respectively; p = 0.697). In contrast, the frequency of infection for *C. perfringens* was higher in CO patients compared with those of HCFO (18.3% and 41.3%, respectively, p = 0.004); this same distribution was observed for tox_*C. difficile* (53.7% and 59.4%, respectively, p = 0.032) (Fig. 1a).

### Statistical associations

In addition, the distribution of infections according to the stay (HCFO and CO) and the hospital of origin of the patients (HCC-1 and HCC-2) was determined. At the HCFO level, infections were higher for the three species in the patients from the HCC-1 healthcare center compared to HCC-2, the distribution observed for *C. difficile* being statistically significant (p = 0.0250) (Fig. 1b). For CO patients, *C. difficile* infections were higher in HCC-2 compared to HCC-1 (p = 0.2285), in contrast to *C. perfringens* whose infections were higher than those observed in HFCO. In community patients from HCC-1, the frequency of occurrence was higher than that observed for HCC-2 (p = 0.2810). A similar pattern was found for the distribution of tox_*C. difficile* between hospital centers which was statistically significant (p = 0.0001) (Fig. 1c).

**Fig. 2:**
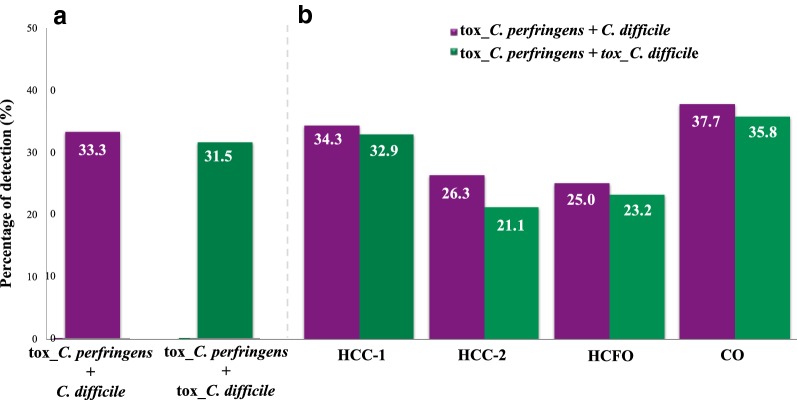
Co-infection frequency between the two evaluated species. **a** In the global population and **b** by Health care center (HCC) and population. *HCFO* Health Care Facility-Onset, *CO* Community Onset)

**Fig. 3:**
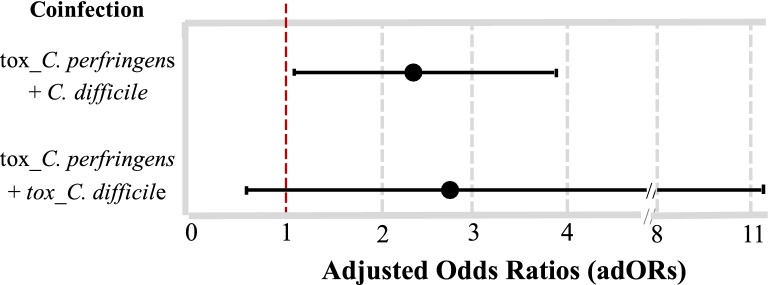
Diagram of strength of association between Clostridial species evaluated. Odds ratios (OR), and their corresponding 95% confidence intervals, which indicate the strength of association between both species according with the type of infection

The evaluation of the coinfection frequencies between the two species evaluated revealed a global percentage of 33.3% for the case of tox_*C. perfringens* + *C. difficile* and 31.5% for tox_*C. perfringens* + tox_*C. difficile* (Fig. 2a). The analysis by HCC and by population, showed that the coinfection frequencies ranged between 21.1% for tox_*C. perfringens* + tox_*C. difficile* in HCC-2, and up to 37.7% for tox_*C. perfringens* + *C. difficile* in CO (Fig. 2b). The results of the OR between the clostridial infections with HCC and stay, showed a positive association for community patients and tox_*C. perfringens* infections (AdOR: 2.69 CI 95% 1.35–5.35). In contrast, a minor association was observed between HCC-2 and tox_*C. difficile* infection (AdOR: 0.14 CI 95% 0.04–0.46) (Table 1). In the same context, AdORs were calculated by the association between the types of coinfections present in the populations evaluated; only positive association was observed for the combination of *C. perfringens* and *C. difficile* (AdOR: 2.05 CI 95% 1.07–3.93) (Fig. 3).

**Table 1:** Logistic regression modeling showing the relationship between a positive result for infection and the hospital and acquisition of infection

	*C. perfringens* infection			*C. difficile* infection			Toxigenic *C. difficile* infection		
	AdOR	[95% CI]	*p*	AdOR	[95% CI]	*p*	AdOR	[95% CI]	*p*
Health care center									
HCC-1	Reference			Reference			Reference		
HCC-2	0.50	[0.17–1.45]	0.206	0.59	[0.26–1.34]	0.208	*0.14*	*[0.04*–*0.46]*	0.001
Service									
HCFO	Reference			Reference			Reference		
CO	*2.69*	*[1.35*–*5.35]*	0.005	0.76	[0.40–1.44]	0.413	1.98	[0.66–5.94]	0.221

Adjusted OR by population and health-care center

Italic values indicate significance of *p* value

## Discussion

The present study identified the frequency of *C. difficile* (general and tox_ *C. difficile*), but also tox_*C. perfringens* in two HCCs of Bogotá, Colombia through molecular detection [5]. It is important to note that previous reports indicate frequent development of diarrhea in individuals in whom the microbiota has been altered by the effect of antimicrobial agents, favoring the proliferation of pathogens such as those belonging to the genus *Clostridium* [14]. This could explain the presence of tox_*C. perfringens* in HCFO diarrhea. However, the results reported in this study indicate greater frequency of infection in patients with CO diarrhea (Fig. 1), which could be related either to the established association of infection by this species with diarrheal disease caused by food poisoning [4], or with the presence of some other factor that could be involved with the development of dysbiosis and acquiring the role of a pathogen [14]. Although, due to the lack of clinical and sociodemographic information of the individuals included in this study, it is not possible to establish an association of causality between the presence of *C. perfringens* or *C. difficile* in patients with diarrhea in Colombia. We found interesting associations between *C. perfringens* and *C. difficile* (AdOR: 2.05 CI 95% 1.07–3.93) (Fig. 3). This suggests that coinfection plays a relevant role in the population. Future studies should consider both species in terms of clinical and sociodemographic data which might provide novel insights regarding the impact of both species in a particular population.

One limitation of our study was the inability to recover the isolates and conduct molecular characterization using genome sequencing or Multilocus Sequence Typing. Therefore, we encourage the scientific community to develop novel studies in the region aimed at unraveling the molecular features of these two species. The findings herein identified represent a baseline about the high coexistence of these two Clostridial species towards depicting the epidemiological panorama in the country and Latin-America.

## Data Availability

All the data are within the manuscript.
